# 2,2-Dichloro-1-(4-methyl­phen­yl)ethanone

**DOI:** 10.1107/S1600536811000134

**Published:** 2011-01-12

**Authors:** Ping-An Wang, Jun-Ping Gao, Peng Liu

**Affiliations:** a Department of Chemistry, School of Pharmacy, Fourth Military Medical University, Changle West Road 17, 710032 Xi-An, People’s Republic of China

## Abstract

The mol­ecule of the title compound, C_9_H_8_Cl_2_O, is almost planar: the dihedral angle between the benzene ring and the plane defined by the carbonyl O and ethane C atoms is 15.5 (2)°. The crystal packing is stabilized by inter­molecular C—H⋯O hydrogen bonds.

## Related literature

For the preparation, see: Aston *et al.* (1943 ▶); Terent’ev *et al.* (2004 ▶). For synthetic use of the title compound and mandelic acid derivatives, see: Schiffers & Bolm (2008 ▶); Blay *et al.* (2006 ▶).
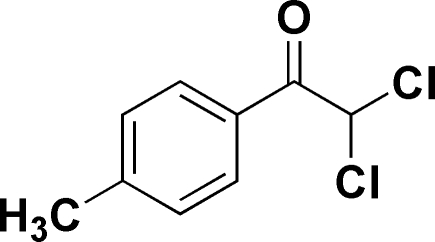


## Experimental

### 

#### Crystal data

C_9_H_8_Cl_2_O
*M* *_r_* = 203.05
Monoclinic, 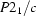

*a* = 6.650 (5) Å
*b* = 9.959 (7) Å
*c* = 14.475 (11) Å
β = 92.921 (9)°
*V* = 957.4 (12) Å^3^
*Z* = 4
Mo *K*α radiation
μ = 0.63 mm^−1^
*T* = 296 K
0.32 × 0.26 × 0.14 mm

#### Data collection

Bruker SMART APEX CCD area-detector diffractometer
Absorption correction: multi-scan (*SADABS*; Bruker, 2005 ▶) *T*_min_ = 0.826, *T*_max_ = 0.920
4496 measured reflections
1694 independent reflections
874 reflections with *I* > 2σ(*I*)
*R*_int_ = 0.061

#### Refinement

*R*[*F* ^2^ > 2σ(*F* ^2^)] = 0.054
*wR*(*F* ^2^) = 0.155
*S* = 1.03
1694 reflections
110 parameters
H-atom parameters constrained
Δρ_max_ = 0.21 e Å^−3^
Δρ_min_ = −0.27 e Å^−3^

### 

Data collection: *APEX2* (Bruker, 2008 ▶); cell refinement: *SAINT* (Bruker, 2008 ▶); data reduction: *SAINT*; program(s) used to solve structure: *SHELXS97* (Sheldrick, 2008 ▶); program(s) used to refine structure: *SHELXL97* (Sheldrick, 2008 ▶); molecular graphics: *SHELXTL* (Sheldrick, 2008 ▶); software used to prepare material for publication: *Mercury* (Macrae *et al.*, 2006 ▶) and *ORTEP-3* (Farrugia, 1997 ▶).

## Supplementary Material

Crystal structure: contains datablocks I, global. DOI: 10.1107/S1600536811000134/bt5447sup1.cif

Structure factors: contains datablocks I. DOI: 10.1107/S1600536811000134/bt5447Isup2.hkl

Additional supplementary materials:  crystallographic information; 3D view; checkCIF report

## Figures and Tables

**Table 1:** Hydrogen-bond geometry (Å, °)

*D*—H⋯*A*	*D*—H	H⋯*A*	*D*⋯*A*	*D*—H⋯*A*
C4—H4⋯O1^i^	0.93	2.58	3.42	150

Symmetry code: (i) 
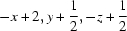
.

**Fig. 1.**
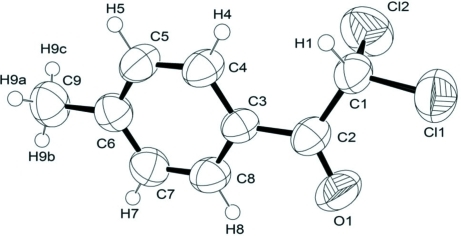
The molecular structure of the title compound, showing the atom-labelling scheme. Displacement ellipsoids are drawn at the 50% probability level. Hydrogen atoms are drawn as spheres of arbitrary radius.

## supplementary crystallographic information

### Comment

The title compound, 2,2-Dichloro-1-(4-methylphenyl)ethanone, was obtained by chloration of 1-(4-methylphenyl)ethanone with concentrated hydrochloride and aqueous hydroperoxide in hot ethanol (Terent'ev *et al.*, 2004) and it was used for the preparation of substituted mandelic acid and derivatives.

In the title compound, C_9_H_8_Cl_2_O, the lengthes of two C—Cl bonds are different, the distance of C1—Cl1 is 1.757 Å, otherwise, the distance of C1—Cl2 is 1.762 Å. The molecule is nearly planar, the dihedral angle between the phenyl ring and the plane defined by O1, C2 and C1 is 15.5°. The packing of molecules in the crystal structure is stabilized by intermolecular C—H···O hydrogen bonds.

### Experimental

To a stirred hot mixed aqueous concentrated hydrochloride and ethanol solution (300 cm^3^) of commercially available 1-*p*-tolylethanone (13.42 g, 0.10 mol) added dropwise aqueous hydropeoxide (35% wt in water, 0.272 mol), and the mixture was stirred for 30 min at 90–100°C. The solution was cooled to room temperature and diluted by addtion of water (300 cm^3^), it was extracted by ether (2× 400 cm^3^), the combined organic layer was washed with 1 *M* NaOH (100 cm^3^), water (100 cm^3^), brine (2× 110 cm^3^), and dried over Na_2_SO_4_. Evaporation of the solvent afforded the title compound as a light yellow oli (19.8 g, 97%), which was solidfied as a pale block after24 h at room temperature. The melting point and the spectroscopic data of the title compound were consisted with the reported literature (Terent'ev *et al.*, 2004).

### Refinement

All H atoms were placed in calculated positions and refined as riding, with C—H = 0.93–0.98Å and with *U*_iso_(H) = 1.2*U*_eq_(C).

### Crystal data

**Table:**

C_9_H_8_Cl_2_O	*F*(000) = 416
*M**_r_* = 203.05	*D*_x_ = 1.409 Mg m^−^^3^
Monoclinic, *P*2_1_/*c*	Mo *K*α radiation, λ = 0.71073 Å
*a* = 6.650 (5) Å	θ = 2.5–25.1°
*b* = 9.959 (7) Å	µ = 0.63 mm^−^^1^
*c* = 14.475 (11) Å	*T* = 296 K
β = 92.921 (9)°	Block, colorless
*V* = 957.4 (12) Å^3^	0.32 × 0.26 × 0.14 mm
*Z* = 4	

### Data collection

**Table:**

Bruker SMART APEX CCD area-detector diffractometer	1694 independent reflections
Radiation source: fine-focus sealed tube	874 reflections with *I* > 2σ(*I*)
graphite	*R*_int_ = 0.061
φ and ω scans	θ_max_ = 25.1°, θ_min_ = 2.5°
Absorption correction: multi-scan (*SADABS*; Bruker, 2005)	*h* = −7→7
*T*_min_ = 0.826, *T*_max_ = 0.920	*k* = −11→9
4496 measured reflections	*l* = −17→15

### Refinement

**Table:**

Refinement on *F*^2^	Primary atom site location: structure-invariant direct methods
Least-squares matrix: full	Secondary atom site location: difference Fourier map
*R*[*F*^2^ > 2σ(*F*^2^)] = 0.054	Hydrogen site location: inferred from neighbouring sites
*wR*(*F*^2^) = 0.155	H-atom parameters constrained
*S* = 1.03	*w* = 1/[σ^2^(*F*_o_^2^) + (0.0679*P*)^2^] where *P* = (*F*_o_^2^ + 2*F*_c_^2^)/3
1694 reflections	(Δ/σ)_max_ < 0.001
110 parameters	Δρ_max_ = 0.21 e Å^−^^3^
0 restraints	Δρ_min_ = −0.27 e Å^−^^3^

### Special details

**Table:**

Geometry. All e.s.d.'s (except the e.s.d. in the dihedral angle between two l.s. planes) are estimated using the full covariance matrix. The cell e.s.d.'s are taken into account individually in the estimation of e.s.d.'s in distances, angles and torsion angles; correlations between e.s.d.'s in cell parameters are only used when they are defined by crystal symmetry. An approximate (isotropic) treatment of cell e.s.d.'s is used for estimating e.s.d.'s involving l.s. planes.
Refinement. Refinement of *F*^2^ against ALL reflections. The weighted *R*-factor *wR* and goodness of fit *S* are based on *F*^2^, conventional *R*-factors *R* are based on *F*, with *F* set to zero for negative *F*^2^. The threshold expression of *F*^2^ > σ(*F*^2^) is used only for calculating *R*-factors(gt) *etc*. and is not relevant to the choice of reflections for refinement. *R*-factors based on *F*^2^ are statistically about twice as large as those based on *F*, and *R*- factors based on ALL data will be even larger.

### Fractional atomic coordinates and isotropic or equivalent isotropic displacement parameters (Å^2^)

**Table:**

	*x*	*y*	*z*	*U*_iso_*/*U*_eq_	
Cl1	1.21054 (15)	0.84106 (11)	0.13668 (8)	0.0912 (5)	
Cl2	0.79412 (19)	0.87565 (15)	0.07241 (8)	0.1147 (6)	
O1	0.9705 (4)	0.6849 (3)	0.2551 (2)	0.0966 (10)	
C1	0.9678 (5)	0.8856 (4)	0.1682 (2)	0.0670 (10)	
H1	0.9707	0.9780	0.1915	0.080*	
C2	0.8918 (5)	0.7929 (4)	0.2428 (2)	0.0627 (9)	
C3	0.7161 (5)	0.8368 (3)	0.2934 (2)	0.0562 (9)	
C4	0.6500 (5)	0.9690 (4)	0.2946 (2)	0.0662 (10)	
H4	0.7173	1.0350	0.2629	0.079*	
C5	0.4837 (5)	1.0019 (4)	0.3433 (3)	0.0711 (10)	
H5	0.4403	1.0907	0.3436	0.085*	
C6	0.3800 (5)	0.9072 (4)	0.3915 (2)	0.0662 (10)	
C7	0.4492 (6)	0.7764 (4)	0.3911 (3)	0.0747 (11)	
H7	0.3823	0.7111	0.4238	0.090*	
C8	0.6142 (6)	0.7406 (4)	0.3439 (3)	0.0691 (10)	
H8	0.6588	0.6521	0.3453	0.083*	
C9	0.1955 (5)	0.9460 (5)	0.4417 (3)	0.0876 (13)	
H9A	0.2314	1.0114	0.4884	0.131*	
H9B	0.1410	0.8679	0.4701	0.131*	
H9C	0.0965	0.9834	0.3985	0.131*	

### Atomic displacement parameters (Å^2^)

**Table:**

	*U*^11^	*U*^22^	*U*^33^	*U*^12^	*U*^13^	*U*^23^
Cl1	0.0822 (7)	0.0892 (9)	0.1049 (9)	0.0029 (6)	0.0303 (6)	−0.0065 (6)
Cl2	0.1093 (9)	0.1452 (13)	0.0881 (9)	0.0030 (8)	−0.0091 (7)	0.0200 (7)
O1	0.0959 (19)	0.0598 (18)	0.137 (3)	0.0230 (16)	0.0350 (18)	0.0209 (17)
C1	0.072 (2)	0.050 (2)	0.080 (3)	−0.0003 (18)	0.019 (2)	−0.0037 (18)
C2	0.065 (2)	0.049 (2)	0.075 (2)	0.002 (2)	0.0035 (19)	−0.0052 (19)
C3	0.0566 (19)	0.046 (2)	0.065 (2)	−0.0002 (17)	0.0016 (17)	0.0010 (17)
C4	0.062 (2)	0.049 (2)	0.088 (3)	−0.0012 (19)	0.0076 (19)	0.0019 (18)
C5	0.067 (2)	0.058 (3)	0.088 (3)	0.012 (2)	0.007 (2)	0.000 (2)
C6	0.063 (2)	0.073 (3)	0.063 (2)	0.000 (2)	0.0003 (18)	0.001 (2)
C7	0.081 (3)	0.068 (3)	0.076 (3)	−0.007 (2)	0.016 (2)	0.007 (2)
C8	0.080 (2)	0.051 (2)	0.076 (3)	0.000 (2)	0.003 (2)	0.0040 (19)
C9	0.070 (2)	0.113 (4)	0.081 (3)	0.009 (2)	0.014 (2)	0.002 (2)

### Geometric parameters (Å, °)

**Table:**

Cl1—C1	1.756 (4)	C5—C6	1.378 (5)
Cl2—C1	1.761 (4)	C5—H5	0.9300
O1—C2	1.206 (4)	C6—C7	1.382 (5)
C1—C2	1.527 (5)	C6—C9	1.508 (5)
C1—H1	0.9800	C7—C8	1.369 (5)
C2—C3	1.476 (5)	C7—H7	0.9300
C3—C4	1.388 (5)	C8—H8	0.9300
C3—C8	1.401 (5)	C9—H9A	0.9600
C4—C5	1.380 (5)	C9—H9B	0.9600
C4—H4	0.9300	C9—H9C	0.9600
			
C2—C1—Cl1	111.9 (3)	C4—C5—H5	119.0
C2—C1—Cl2	107.2 (2)	C5—C6—C7	117.9 (3)
Cl1—C1—Cl2	110.86 (19)	C5—C6—C9	120.6 (4)
C2—C1—H1	109.0	C7—C6—C9	121.5 (4)
Cl1—C1—H1	109.0	C8—C7—C6	121.5 (4)
Cl2—C1—H1	109.0	C8—C7—H7	119.2
O1—C2—C3	122.6 (3)	C6—C7—H7	119.2
O1—C2—C1	119.2 (3)	C7—C8—C3	120.2 (4)
C3—C2—C1	118.1 (3)	C7—C8—H8	119.9
C4—C3—C8	118.7 (3)	C3—C8—H8	119.9
C4—C3—C2	123.1 (3)	C6—C9—H9A	109.5
C8—C3—C2	118.2 (3)	C6—C9—H9B	109.5
C5—C4—C3	119.6 (3)	H9A—C9—H9B	109.5
C5—C4—H4	120.2	C6—C9—H9C	109.5
C3—C4—H4	120.2	H9A—C9—H9C	109.5
C6—C5—C4	122.0 (4)	H9B—C9—H9C	109.5
C6—C5—H5	119.0		
			
Cl1—C1—C2—O1	−19.0 (4)	C2—C3—C4—C5	179.7 (3)
Cl2—C1—C2—O1	102.8 (3)	C3—C4—C5—C6	0.1 (6)
Cl1—C1—C2—C3	164.5 (2)	C4—C5—C6—C7	1.0 (5)
Cl2—C1—C2—C3	−73.7 (3)	C4—C5—C6—C9	−178.3 (3)
O1—C2—C3—C4	166.2 (4)	C5—C6—C7—C8	−0.7 (5)
C1—C2—C3—C4	−17.4 (5)	C9—C6—C7—C8	178.5 (3)
O1—C2—C3—C8	−12.5 (5)	C6—C7—C8—C3	−0.7 (5)
C1—C2—C3—C8	163.8 (3)	C4—C3—C8—C7	1.9 (5)
C8—C3—C4—C5	−1.6 (5)	C2—C3—C8—C7	−179.4 (3)

### Hydrogen-bond geometry (Å, °)

**Table:**

*D*—H···*A*	*D*—H	H···*A*	*D*···*A*	*D*—H···*A*
C4—H4···O1^i^	0.93	2.58	3.42	150

Symmetry codes: (i) −*x*+2, *y*+1/2, −*z*+1/2.
